# Tricuspid Valve Endocarditis Due to Methicillin-Resistant *Staphylococcus aureus* in a Previously Healthy Young Patient without a Drug Abuse History: A Case Report and a Review of the Literature

**DOI:** 10.3390/idr15030033

**Published:** 2023-06-12

**Authors:** Nataša Andrijašević, Martina Perešin Vranjković, Karolina Dobrović, Irina Pristaš, Saša Andrašević, Arjana Tambić Andrašević

**Affiliations:** 1 University Hospital for Infectious Disease Fran Mihaljevic, Mirogojska 8, 10000 Zagreb, Croatia; mperesin@bfm.hr (M.P.V.); sandrasevic@bfm.hr (S.A.); atambic@bfm.hr (A.T.A.); 2 University Hospital Dubrava, Avenija Gojka Šuška 6, 10000 Zagreb, Croatia; 3 School of Dental Medicine, University of Zagreb, 10000 Zagreb, Croatia; 4 Department of Clinical Microbiology, University of Applied Health Sciences in Zagreb, 10000 Zagreb, Croatia

**Keywords:** right-sided infective endocarditis (RSIE), methicillin-resistant *Staphylococcus aureus*, MRSA, non-intravenous drug abusers, no medical burden

## Abstract

Right-sided infective endocarditis due to methicillin-resistant *Staphylococcus aureus* (MRSA) is strongly associated with intravenous drug abuse, congenital heart disease, or previous medical treatment and is rare in healthy patients without a history of drug abuse. Here, we present a case of an 18-year-old male with no drug abuse history and no medical burden who was diagnosed with MRSA tricuspid valve endocarditis. Due to initial symptoms which indicated community-acquired pneumonia and radiological finding of interstitial lesions, empiric therapy with ceftriaxone and azithromycin was started. After the detection of Gram-positive cocci in clusters in several blood culture sets, endocarditis was suspected, and flucloxacillin was added to the initial therapy. As soon as methicillin resistance was detected, the treatment was switched to vancomycin. Transesophageal echocardiography established the diagnosis of right-sided infective endocarditis. A toxicological analysis of hair was carried out, and no presence of narcotic drugs was found. After six weeks of therapy, the patient was fully recovered. Exceptionally, tricuspid valve endocarditis can be diagnosed in previously healthy people who are not drug addicts. As the clinical presentation commonly resembles a respiratory infection, a misdiagnosis is possible. Although MRSA rarely causes community-acquired infections in Europe, clinicians should be aware of this possibility.

## 1. Introduction

Infectious endocarditis (IE) is a widely recognized and extensively studied clinical syndrome characterized by infection of the heart’s inner lining and valves. This severe and potentially life-threatening condition is associated with high morbidity and mortality rates, making it a critical concern for medical professionals and patients as well [1,2]. Cardiac vegetations are most commonly found in the left heart, on mitral (41%), and aortic (37.6%) valves with *S. aureus* as the most often isolated pathogen [1,2,3]. Despite advances in clinical diagnosis and significant improvement of early recognition and aggressive intervention, the treatment of this syndrome often results in failure. The patients diagnosed with IE continue to face a significant risk of mortality and the in-hospital mortality rate is high, accounting for 15% to 30% [4]. Right-sided infective endocarditis (RSIE) is rarely diagnosed and accounts for a notably smaller proportion of patients, from 5% to 10% of all IE [5,6,7,8]. It mostly involves the tricuspid valve with a frequency of 61% and a less common pulmonary valve with a frequency of 23%. Vegetations on the endocardium are infrequent and account for 7.6% of cases [7]. RSIE occurs more often in males than in females and is strongly associated with intravenous drug use (IVDU), as well as intracardiac devices, such as pacemakers, implantable cardioverter defibrillators, or cardiac loop recorders. The previous or current presence of central venous catheters, hemodialysis, congenital heart disease, and previous surgical treatment also raises the risk of developing RSIE [5,6,7,8].

Patients with RSIE usually present with elevated body temperature associated with chills, anorexia, and weight loss. These symptoms can often be followed by a range of other nonspecific symptoms, such as headache, malaise, joint and muscle pain, night sweats, and abdominal pain. Dyspnea and cough are often concomitant symptoms, as septic pulmonary emboli are common among patients with RSIE. Patients often present with pleuritic chest pain and occasionally hemoptysis [9]. Overall mortality for RSIE is high and counts for 5–15% [8]. Patients without risk factors account for various proportions of all patients with RSIE, ranging from 1.7% to 20% [5,7,10,11]. In some countries, such as Slovenia, where IVDU have free sterile needles available, and India which has a small proportion of intravenous drug addicts, IVDU does not present a major risk factor for the development of RSIE [7,10]. In RSIE, *S. aureus* is a predominant pathogen responsible for 50–90% of cases, with an increased incidence of methicillin-resistant *S. aureus* (MRSA) [2,6,8,11]. MRSA most likely forms vegetations on the tricuspid valve in IVDU but there are few individual case reports of MRSA endocarditis in non-IVDU individuals with a burdened medical history [5,11,12,13]. The fact that colonization with MRSA increases the risk of developing MRSA-related infections is well known. In persons colonized with MRSA, bacteremia caused by this agent is most often seen in immunosuppressed patients and patients with diabetes mellitus [14]. It should be pointed out that nasal carriage of MRSA has increased in recent years, particularly in the United States. In some parts of the country, children’s MRSA colonization rate is as high as 9.2% [15]. Moreover, in the United States, the proportion of outpatient MRSA infections increased in just a few years, from 29% in 2001–2002 year to 64% in 2003–2004 [16]. According to available Canadian studies, although the prevalence rate of MRSA has increased year by year, it has not reached close to the rate in the USA, and the prevalence in hospitals across the country is 7.2% [17]. The prevalence of outpatient MRSA infections in Europe is significantly lower than in the United States, with an overall prevalence rate of 15.1%, varying from 0% in the north of Europe to 29% in the south of the European subcontinent [18]. 

Following an extensive review of the relevant literature, it could be concluded that there are just a few reports of RSIE in healthy adults without any medical burdens [5,7,9,10]. Additionally, it can be noticed that the incidence of RSIE caused by MRSA is relatively low. Our analyses revealed a total of 16 papers reporting on this syndrome, collectively presenting data on 22 patients diagnosed with this condition. Of the patients included in these reports, majority (16/22, 72.7%) had a pre-existing medical condition that was identified as a significant risk factor for developing RSIE. Except for IVDU, following were the previous medical conditions described as a risk factor for developing RSIE: previous MRSA bacteremia, previous MRSA endocarditis, skin infections caused by MRSA, MRSA osteomyelitis, frequent parenteral administration of saline, prosthetic valve, ventricular septal defect, chronic hemodialysis, MRSA pneumonia, intravenous cannula and the presence of an intracardiac device. Following this, colonization with MRSA was found to be the second most common risk factor, affecting 40.9% (9/22) of patients. Interestingly, IVDU was identified as the third most prevalent risk factor, with 36.4% (8/22) of patients reporting this behavior as a potential contributing factor (Table 1). Despite our extensive research efforts, we found that data on MRSA RSIE in individuals without a history of IVDU or any other known medical risk factors were exceedingly rare, with only one such case report identified in Japan [19]. As such, further research in this area is necessary to better understand the pathophysiology and epidemiology of this condition, particularly in the absence of traditional risk factors. 

In the case of outpatient infections caused by MRSA, Panton Valentine (PV) leukocidin should also be mentioned. Panton Valentine (PV) leukocidin is a pore-forming exotoxin produced by the bacterium *S. aureus*. In vitro, studies have shown that this exotoxin leads to the death of polymorphonuclear cells either by necrosis or apoptosis, depending on the concentration of the toxin itself. PV leukocidin has the ability to form pores in the outer membrane of human mitochondria. It was also detected in the tissue of patients who died of necrotizing pneumonia caused by *S. aureus*. From the above, it can be concluded that this staphylococcal exotoxin can be the cause of lung tissue necrosis in vivo, thus creating a clinical picture of necrotizing pneumonia [31]. The presence of Panton-Valentine leukocidin as a significant virulent factor in community-acquired (CA) MRSA infections also varies between continents. In the United States, PV leukocidin prevalence is significantly high and accounts for more than 50%, while in Europe, it accounts for 24.9% [15,18].

Here, we describe a case of a previously healthy young man who developed RSIE due to MRSA. The patient had a morphologically regular tricuspid valve and had no history of intravenous drug abuse. This case underscores the importance of early diagnosis and prompt treatment for infective endocarditis and indicates the possibility of intracardiac MRSA infection even in patients without any known previous risk factors.

## 2. Case Presentation

An 18-year-old previously healthy young man without a history of IVDU was admitted to the University Hospital for Infectious Diseases Dr Fran Mihaljevic in Zagreb, Croatia. Upon arrival, he complained of an elevated body temperature of up to 39.5 °C, occasionally with chills. The patient also complained of generalized myalgia, malaise, non-productive dry cough, and mild right-sided chest pain. He denied dyspnea. The symptoms had persisted for the last 10 days. The patient regularly smoked 15 cigarettes a day, occasionally drank small amounts of alcohol, and did not take any medications. He had no history of any previous medical conditions. Due to the COVID-19 pandemic and the lockdown, he has not attended school or worked out in the gym for several months, but before the lockdown, he was a high school student and occasionally worked out in the gym. He also denied any drug abuse and he did not recently travel abroad. The patient disclosed no multiple sexual engagements with men and reported a sustained long-term monogamous partnership. 

On admission day, the patient was feverish (Tax 37.5 °C after antipyretics) with pale skin. The patient’s blood pressure was 120/80 mm Hg, heart rate was 120 beats per minute, and blood oxygen level was measured to be 94%. Whole body skin examination did not reveal eczema-type lesions, furuncles, or carbuncles, which could be considered as a possible source of bacterial infection. Moreover, any skin signs of septic emboli, or skin marks from venipuncture were not present. Clinical signs that would point to meningism were also negative and the patient did not show any neurological deficits. His general condition appeared to be moderately compromised. Laboratory blood analyses indicated the presence of a probable bacterial infection and revealed elevated blood values of leukocytes with the domination of neutrophilic granulocites and reactive lymphopenia. C-reactive protein, procalcitonin, lactate dehydrogenase, ferritin, and fibrinogen-activity were also elevated. Significant leukocytosis with neutrophilia was also observed. Additionally, anemia was present, while the values of creatinine, blood urea nitrogen, and transaminases fell within the normal range. D-dimers showed elevated levels, whereas the value of creatinine kinase fell within the reference interval (Table 2).

The polymerase chain reaction test ruled out COVID-19, and serology for it also confirmed negative results. Biochemical urinalysis results were in the normal range. As the patient’s cough was not productive, microbiological analysis of the sputum was not performed. The flow cytometry was performed and the result showed an increased number of NK cells, activated T-lymphocytes, and the ratio of CD8+/CD38+ lymphocytes. The values of CD3+ T-lymphocytes, CD-19+ B-lymphocytes, CD3+/CD4+ helper lymphocytes, CD3+/CD8+ cytotoxic T-lymphocytes, the absolute number of CD4+ T-lymphocytes, as well as the value of the CD4+/CD8+ t-lymphocyte ratio, were within the normal range. The values of total and specific proteins (IgG, IgA, and IgM) in the blood, values of total kappa and total lambda light chains, and kappa/lambda ratio were within the normal range, and monoclonal M protein in serum was not detected via immunofixation. Electrophoresis of serum proteins showed reduced values of albumin and elevated values of Alpha 1 and Alpha 2 globulin (Table 2). Beta and gamma globulins were within normal range. Biomarkers for connective tissue diseases (ANA screen, and dsDNA), vasculitis (ANCA-PR3s, and ANCA-MPOs), rheumatoid arthritis (RF IgA, RF IgM, and CCP), and autoimmune liver diseases (AMA-M2, and LKM-1) were performed and results for all were negative. The electrocardiological findings were pathological and showed sinus tachycardia (100/min) with a prolonged QTc interval of 0.46 ms. Due to the laboratory results that indicated bacterial infection and chest radiography findings of bilateral and interstitial lesions in both lungs, the initial diagnosis of CA bacterial pneumonia was established. The *Legionella* urine antigen test was performed and the result for it was negative. Empirical treatment for CA pneumonia with parenterally administered ceftriaxone (2 g once a day ) and azithromycin (500 mg once a day IV) was initiated immediately upon hospital admission. As soon as the growth of Gram-positive cocci in clusters was reported in two sets of blood cultures taken on admission day, the diagnosis of infective endocarditis was suspected. Consequently, empiric therapy with flucloxacillin (2 g qid IV) was added to the initial empirical antibiotic treatment. On the second day of hospital treatment, the patient developed bilateral pleural effusion (depths of 2 cm on the right and 3 cm on the left side of the chest). Soon, the antibiotic susceptibility pattern for *S. aureus* showed resistance to penicillin, flucloxacillin (MIC 32 mg/L), and macrolides, and sensitivity to clindamycin, moxifloxacin, gentamicin, sulfamethoxazole/trimethoprim, rifampicin, tigecycline, and linezolid. A minimal inhibitory concentration of vancomycin was performed and it measured 0.75 mg/L. According to the European Committee for Antimicrobial Susceptibility Testing (EUCAST) breakpoints for the 2020 year, this value was interpreted as sensitive. Following antimicrobial testing results and confirmation of MRSA isolated from several blood culture sets, it was obvious that empiric therapy would not be effective. Consequently, treatment was switched to vancomycin in a recommended daily dose of 1 g twice a day IV over 60 min. The real-time polymerase chain reaction for the PV leukocidin gene was also conducted and the result was negative. After receiving the preliminary blood culture results, surveillance swabs of the nasopharynx, armpits, and groin for multi-resistant pathogens were taken at the department, and the result confirmed that the patient was carrying MRSA, both on his skin and in their nasal passages. The antibiotic sensitivity of this isolate was found to be identical to that of the blood culture isolate. 

**Figure 1:**
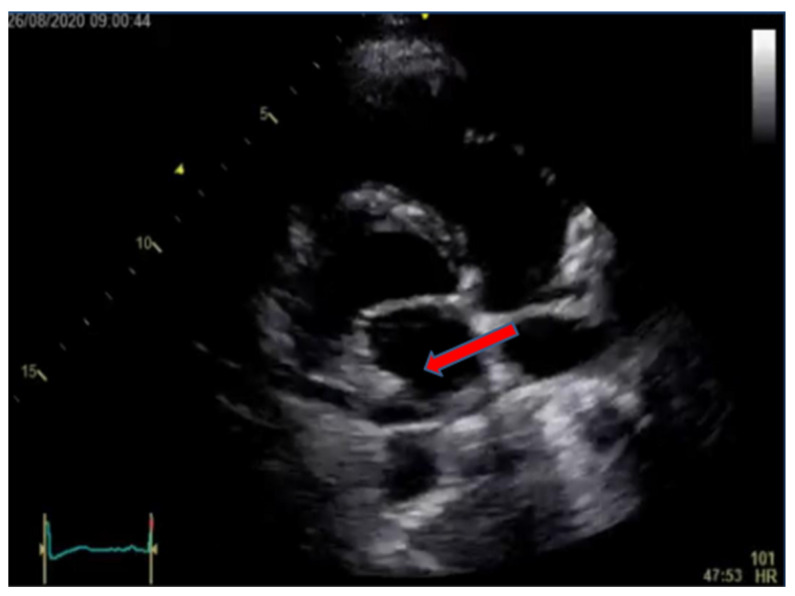
Transesophageal echocardiogram (TEE) showing large tricuspid vegetation on mid-esophageal four-chamber view (arrow showing tricuspid vegetation).

As the diagnosis of probable endocarditis was established, transthoracic echocardiography (TEE) was performed and showed no vegetation on any of the heart valves. Consequently, an indication for transesophageal ultrasonography was established, and the result of this examination showed a floating vegetation, sized 3 × 11 mm, on the anterior leaflet of the tricuspid valve with significant tricuspid regurgitation (3+). There were no signs of vegetation in the mitral, aortic, or pulmonary valve (Figure 1). 

Although the patient denied any history of drug abuse, he was suggested a toxicological hair analysis, which was carried out with his written consent. The result of this analysis ruled out the possibility of drug abuse as the presence of narcotic drugs (amphetamine, methamphetamine, “Ecstasy”, morphine, codeine, heroin, heptanol, and cocaine) was negative. Additionally, the results of the HIV serological testing were negative. According to the vancomycin trough levels in the blood, the initial daily dose of parenteral vancomycin needed to be adjusted to 1 g tid IV over 60 min in order to achieve values that satisfy the intended concentration of vancomycin for this indication. Creatinine and eGFR levels remained in the normal range. 

The patient was hospitalized for the next 6 weeks during which intravenous vancomycin therapy was administered. On his discharge day, TEE was performed and its findings showed a significant reduction in tricuspid regurgitation with milder size vegetation on the tricuspid valve. The patient was discharged in good general condition and remained under regular supervision by the cardiologist and showed no further signs of the disease. 

## 3. Discussion

Here, we presented a rare case of RSIE caused by an MRSA in a young man who had no prior medical history of drug abuse or any other underlying medical conditions. The rarity of this case is underscored by the limited number of documented cases of RSIE caused by MRSA in healthy individuals without any known risk factors. The clinical presentation of RSIE usually consists of a febrile syndrome associated with anemia, cough, and chest pain. The murmur of tricuspid regurgitation as a significant clinical sign does not need to be present. As symptoms of RSIE coincide with the signs of a respiratory infection, it is not unusual to confuse this diagnosis with the diagnosis of community-acquired pneumonia [9]. Given the morphology of the radiological findings, it was challenging to definitively discern whether the changes originated from pneumonic infiltration or were a consequence of the early phase of cardiac decompensation associated with endocarditis. Without any positive anamnestic data for intravenous drug abuse, known heart disease, previously performed surgery, present intravenous catheter, or hemodialysis, the clinician can easily overlook RSIE as a leading diagnosis. While the literature indicates that changes in the skin can be considered as a risk factor for the development of endocarditis, it is noteworthy that the presented patient had intact skin without any apparent abnormalities [32]. In the case presented, the leading diagnosis on the admission day was CA pneumonia and thus empiric antimicrobial therapy was introduced according to the local guidelines for that specific diagnosis. Only after the isolation of *S. aureus* from multiple sets of blood cultures, presence of infective endocarditis was clinically suspected. Since the overall methicillin resistance rate of *S. aureus* in Croatia is about 20%, and considering the fact that community-acquired MRSA prevalence is low and counts for less than 0.5%, it was a logical decision to empirically add flucloxacillin and not vancomycin to the patient’s current therapy for CA pneumonia [33,34]. As soon as antimicrobial susceptibility testing results were completed and methicillin resistance in *S. aureus* was confirmed, therapy was finally switched to targeted intravenous administered vancomycin, following the discontinuation of the previously administered therapy with the other three antibiotics. The dose of intravenous vancomycin still needed fine-tuning in order to achieve satisfactory vancomycin blood concentration levels results. Prolonged MRSA bacteremia without any additional risk factors may not necessarily have a strong statistical association with the diagnosis of IE [35]. However, patients who have MRSA bacteremia along with some other clinical prediction criteria, including prolonged duration of bacteremia, are still considered to be at a higher risk for IE and thus should undergo a clinical evaluation, such as a TEE [4,35]. Following the 2015 European Society of Cardiology Guidelines for the management of infective endocarditis, despite a negative result from a TTE, we decided to conduct another TEE examination [4]. This later TEE examination revealed lesions on the tricuspid valve with significant tricuspid regurgitation. Furthermore, if additional risk factors, such as intravenous drug use, intravascular prosthetic material, skin lesions, or previous medical treatment were present, the leading diagnosis would likely be established more promptly, potentially before subsequent positive blood cultures occur. According to the available guidelines for MRSA-caused endocarditis, the first drug of choice is vancomycin, so the treatment with this antibiotic was started with an initial dose of 1 g twice a day IV over 60 min [2,4,9]. This initial vancomycin dosing regime did not adapt to the patient’s creatinine clearance and age, and vancomycin trough level in blood showed to be lower than the expected values of 15–20 mg/L [36]. Consequently, the dose of administered vancomycin needed to be corrected to 1 g tid IV over 60 min. After this dose correction, satisfactory vancomycin trough values were observed, and the patient started slowly to recover. After a full course of 6 weeks of intravenous therapy, the patient was discharged from hospital treatment and sent for home recovery. 

Because of the obtained results, it can be stated that instead of vancomycin therapy, daptomycin could be considered as a targeted drug of choice. Daptomycin is also approved for the treatment of bloodstream infections, and dosing with daptomycin is simpler than dosing with vancomycin. In the presented patient, therapy with vancomycin was started according to the UpToDate recommendations and the standard practice of the University Hospital for Infectious Diseases [8]. Although both vancomycin and daptomycin have an equal effect on mortality, a retrospective analysis of the data revealed that vancomycin therapy was more likely to result in clinical failure [37]. Ceftaroline could also be used for treating infections caused by MRSA. It has been approved by the FDA for treating bacteremia that accompanies community-acquired pneumonia. However, it is not approved for treating bacteremia originating from other sources [38]. While some smaller retrospective clinical studies have described the successful use of ceftaroline in treating endocarditis, this antibiotic was not considered as a potential therapy due to its sensitivity to vancomycin of the isolated MRSA strain [39,40]. The application of combined ceftaroline–daptomycin therapy is also possible, but indications for its use are not yet well-defined [38,41]. In patients with no prior medical history, the most obvious anamnestic data for RSIE caused by MRSA would be an abuse of intravenous narcotics [1,2,3]. Guided by the fact that users of opioids often provide unreliable data and withhold information about their drug abuse, an additional test of hair strands for drug consumption was performed with the patient’s written consent [42]. The test result was negative which confirmed the patient’s anamnestic data. As the number of new synthetic drugs intended for intravenous administration is increasing daily, with new chemical compounds appearing from time to time around the globe, the possibility that some of these compounds are not included in this toxicological testing cannot be ruled out [43]. Although the patient denied the use of narcotics or any other medical products and no signs of venepuncture were recorded on his skin, it should be considered that a discrete possibility of narcotic abuse still exists, however, not for IV use. 

The incidence of infective endocarditis (IE) in patients with HIV who engage in intravenous drug use (IVDU) is reported to be approximately 6.3%. However, in patients with late-stage HIV infection, the risk of IE increases significantly, with incidence rates being much higher, reaching up to 30% [44]. Despite this correlation, in the patient being presented, the serological HIV test results were negative, and thus the risk factor of HIV-related IE was ruled out. To further investigate and rule out any potential immune-related causes, the patient underwent a series of additional blood tests. It was concluded that diabetes was not a possible risk factor since the blood sugar levels were normal. Laboratory tests also revealed that immunodeficiency was unlikely to be the cause of the infection. The flow cytometry results exhibited distinct patterns consistent with an ongoing bacterial infection. Serum protein electrophoresis findings were observed as a result of acute infective endocarditis and an inflammatory response triggered by the infection. Furthermore, negative biomarker results for connective tissue diseases, vasculitis, rheumatoid arthritis, and autoimmune liver diseases ruled out the possibility of an autoimmune disease as the underlying condition

CA infections caused by MRSA are still scarce in Europe in contrast to the frequency of these infections in the United States, and the presence of PV leukocidin represents an additional risk for the development of MRSA infection [15,16,18]. Though the presented patient had no history of recent travel abroad, MRSA was detected in the patient’s surveillance cultures. Additionally, the MRSA isolated from the patient’s positive blood cultures possessed no gene for the expression of the leukocidin previously mentioned. In addition to endocarditis, other MRSA-related heart infections should also be mentioned, such as MRSA-caused pericarditis. Although infective pericarditis is a rare diagnosis, a case presentation of a patient, who is a smoker and an alcoholic, and who was not previously treated medically, and who developed MRSA-caused pericarditis was described [45]. CA-MRSA infections have been described in several reports in various populations, including military members, prisoners, men who have sex with men, athletes, and members of some ethnic groups [32]. While the patient did not present any anamnestic data indicating risky sexual behavior it is worth noting that the presented patient exercised in the gym before the COVID-19 lockdown. Considering this, the possibility that he was colonized with MRSA in this gym environment could not be ruled out. Previous studies indicate that the total prevalence of *S. aureus* on fitness and gym surfaces is about 38%, with resistance to oxacillin being as high as 52.8% [46]. This indicates that gym environments can be a potential source of MRSA colonization, and highlights the importance of maintaining proper hygiene practices in these spaces. Based on the data that the prevalence of MRSA colonization among athletes is high and that the risk of developing infections caused by MRSA is significantly higher for colonized than for non-colonized athletes, the possibility exists that the presented patient has acquired MRSA colonization in the gym environment at some point in the past [47].

## 4. Conclusions

The rarity of the case highlights the importance of considering that RSIE, although a rare clinical entity, can also develop on an anatomically healthy valve even in patients without a record of past medical interventions or a history of intravenous drug abuse. 

The increasing prevalence of multiple-resistant bacteria in hospital environments also suggests that these bacteria are likely to become more prevalent in outpatient settings. Therefore, it should not be surprising that the RSIE caused by multidrug-resistant bacteria, such as MRSA, can also develop in healthy, non-drug-addicted patients in outpatient settings. These findings emphasize the need for healthcare providers to be vigilant in identifying and treating RSIE, particularly in patients who do not exhibit any obvious risk factors. It is important to conduct thorough assessments and consider the possibility of multidrug-resistant bacteria when treating patients with RSIE. In addition, efforts should be made to prevent the spread of multidrug-resistant bacteria, primarily by reducing the use of antibiotics in both hospital and outpatient settings.

## Figures and Tables

**Table 1:** Overview of available articles describing cases of RSIE caused by MRSA obtained via searching the PubMed database using the terms right-sided endocarditis and MRSA. Keywords were combined with “AND”.

Author, Year [References]	Country	Design	Age Male (M) Female (F)	IVDU	Medical Risk Factors	Record of MRSA Carrier or Previous MRSA Infection	Targeted MRSA Treatment	Heart Operation Performed	In Hospital Death
Morita K 2021 [19]	Japan	Case report	28 M	No	No	No	Van	Yes	No
Yazaki M 2018 [20]	Japan	Case report	26 F	No	Ventricular septal defect	No	Van + Rif + Gen Switch to Dapt + LZD	Yes	No
Haque N Z 2007 [21]	USA	Retrospective case series	18 F	Yes	No	No	Van + Gen	No	No
			67 M	Yes	No	No	Van + Gen	No	No
			44 F	Yes	No	No	Van + Gen	No	No
			41 F	Yes	No	No	Van + Gen Switch to Dapt + Clin	No	No
			36 F	Yes	Previous MRSA endocarditis MRSA skin Abscess	Yes	Van + Rif + Gen	No	No
Saravu K 2012 [22]	India	Case report	57 M	No	Alcohol abuse Multiple treatments of dehydration with saline sol. Iv	Yes	Tec + Rif	No	No
Sundaragiri P R 2015 [23]	USA	Case report	31 M	Yes	A recent episode of MRSA IE	Yes	Van Switch to Dapt + Ceft	Yes	Yes
Galanter K M 2019 [24]	USA	Case report	36 F	Yes	Previous MRSA bacteriemia	Yes	Van Switch to Dapt + Gen Switch to Dapt + Lzd Switch to Lzd + Gen	Yes	No
Souli M 2005 [25]	Greece	Case report	67 M	No	Prosthetic valve Previous MRSA RSIE	Yes	Van + Rif + Gen + Sxt Switch to Tec + Rif + Sxt Switch to Lzd + Rif	No	No
Liu C L 2008 [26]	Taiwan	Case report	86 M	No	ICD DM Chronic hemodialysis	No	Van + Rif Switch to Dapt + Rif Switch to Lzd + FucA Switch to Teico	No, but ICD removed	No
Zainah H 2013 [27]	USA	Case report	24 F	Yes	No	No	Dapt Switch to Ceft	No	No
Villar E 1998 [28]	France	Case report	49 M	No	Ventricular septal defect Skin Thoracic abscess	No	Van	Yes	No
Hirakava N 2004 [29]	Japan	Case report	76 F	No	Chronic hemodialysis MRSA abscess at the site of the dialysis shunt	Yes	Van + Arb + Min	No	yes
Dortet L 2013 [30]	France	Case report	55 F	No	COPD MRSA pneumonia and catheter-associated bacteriemia	Yes	Dapt + Rif Switch to Van + Gn + Lzd	No	No
Antoun M 2020 [14]	USA	Case report	56 M	No	DM Skin MRSA infection	Yes	Van Switch to Dapt + LZD + Ceft	Yes	No
Chesi G 2006 [12]	Spain	Case report	50 M	No	DM Previous MRSA osteomyelitis	Yes	Tec + Stx Switched to Lzd Switch to Van + Rif + Sxt Switch to Quin/Dalf	No	No
Boukhari E 2000 [13]	Saudi Arabia	Case report	3 F	No	DM 10 days inserted iv cannula	No	Van + Gen Switch to Van + Gen + Cip + Rif	Yes	Yes
Revilla A 2021 [5]	Spain	Prospective case study	54 M	No data	Aortic valve replacement	No data	No data	No	No
			22 M	No data	Intravascular catheter	No data	No data	No	No
			58 M	No data	Local infection	No data	No data	No	No

Van—vancomycin, Gen—Gentamycin, Rif—Rifampicin, Lzd—Linezolid, Dapt—Daptomycin, Sxt- Sulpometoxatole-trimethoprim, Quin/Dalf—Quinupristin/Dalfopristine, Cip—Ciprofloxacin, Min—Minocycline, FucA—Fusidic Acid, Clin—Clindamycin, Tec—Teicoplanin, Arb-Aberkacin Sulfate DM—Diabetes mellitus, COPD—Chronic obstructive pulmonary disease, and ICD—intracardiac device.

**Table 2:** Abnormal blood laboratory test results upon admission.

Laboratory Test	Result (H-High/L-Low)	Unit	Reference Interval
**Leucocytes**	20.6 H	×10^9^/L	3.4–9.7
**Erythrocytes**	4.32 L	×10^12^/L	4.34–5.72
**Hemoglobin**	129 L	g/L	138–175
**Hematocrit**	0.380 L	L/L	0.415–0.530
**Neutrophilic granulocytes**	16.1 H	×10^9^/L	2.06–6.49
**Monocytes**	2.9 H	×10^9^/L	0.12–0.84
**Neutrophil granulocytes**	82 H	rel %	44–72
**Lymphocytes**	7.6 L	rel %	20–46
**Monocytes**	14.0 H	rel %	2–12
**CRP**	352.1 H	mg/L	<5
**LDH**	356 H	U/L	127–231
**Procalcitonin**	0.804 H	µg/L	<0.5
**Fibrinogen-activity**	6.4 H	g/L	1.8–3.5
**Ferritin**	1440 H	µg/L	6–320
**D-dimers**	1.45 H	mg/L	<0.5
**CD3-/CD56+** **(NK cells)**	21.0 H	%	0.0–15.0
**CD3+/HLA-Dr+** **(Activated** **T-lymphocytes)**	13.6 H	%	0.0–10
**CD3+/CD38+**	11.0 H	%	0.9–7.0
**Albumin**	32.6 L	g/L	39.5–58.6
**Alfa 1 globulin**	5.0 H	g/L	0.7–2.6
**Alfa 2 globulin**	11.9 H	g/L	4.9–10.2

## Data Availability

The data presented in this study are available on request from the corresponding author. The data are not publicly available due to the protection of patient privacy.
